# An efficient charging strategy for wireless sensor networks based on saturation degree and Enhanced Grey Wolf optimization

**DOI:** 10.1038/s41598-025-19243-9

**Published:** 2025-10-10

**Authors:** P. Neelagandan, S. Balaji

**Affiliations:** Department of Mathematics, Vellore Institute of Technology, 600127 Chennai, Tamil Nadu India

**Keywords:** Wireless chargers, Degree of saturation, Enhanced Grey Wolf algorithm, Optimal position of chargers, Energy science and technology, Engineering, Mathematics and computing

## Abstract

Wireless sensor networks play a vital role in a wide range of modern applications, from environmental monitoring to industrial automation. A key challenge in maintaining the long-term functionality of these networks lies in effective energy management, where recharging sensors is often more practical and economical than frequent battery replacement. One critical aspect of this process is the optimal placement of chargers to ensure maximum sensor coverage while minimizing deployment costs. This paper presents a hybrid optimization framework that combines graph-theoretical concepts–specifically the Degree of Saturation approach–with the Enhanced Grey Wolf Optimization algorithm to solve the charger placement problem in WSNs. The Degree of Saturation method identifies independent groups of sensors to reduce the number of chargers required, while Enhanced Grey Wolf Algorithm determines their optimal spatial positions to ensure efficient energy replenishment. Extensive simulations demonstrate the superiority of the proposed method over conventional techniques. Compared to wavelet-based approaches such as Haar (83%), Daubechies 2 (85%), Biorthogonal (86%), and Symlets 8 (85%), as well as evolutionary algorithms like Raindrop (87%) and Blackhole (91%), the proposed Enhanced Grey Wolf Optimization-based method achieves a significantly higher efficiency of 97%. These results highlight the robustness and effectiveness of the proposed approach for real-world Wireless sensor networks deployment.

## Introduction

Sensors play a vital role in various fields like monitoring cattle in dairy farming, tracking potential threats at country borders, securing private areas, and even preventing natural disasters^1^. Given their widespread use, sensors have become crucial in our daily lives^2^. A sensor node handles communication within the network^3^, and its lifespan depends on its battery power. Recent studies have explored energy optimization through bio-inspired and swarm intelligence algorithms^4,5^, and wireless energy transfer strategies have also been investigated for improved sensor network efficiency^6^. Furthermore, the application of meta-heuristic algorithms for optimization problems has gained significant attention due to their ability to efficiently handle complex problems in diverse fields^7,8^. Deployment can be either deterministic^9^ or random^10^. In deterministic deployment, sensor locations are predefined, making recharging simpler. However, in random deployment scenarios, where sensor positions are unpredictable, efficient placement of chargers becomes a challenging task.

 The core aim of this study is to optimize the charger placement in wireless rechargeable sensor networks (WRSNs) such that every sensor is recharged using the fewest number of chargers possible, while ensuring full coverage. This problem becomes more critical in large-scale WRSNs where redundant charging leads to increased energy consumption and system inefficiency.

**Motivation:** While several evolutionary algorithms such as Particle Swarm Optimization (PSO)^11^, Genetic Algorithm (GA)^12^, and the Improved Firefly Algorithm (IFA)^13^ have been proposed for charger placement, they primarily focus on placement optimization without first minimizing the number of chargers required. As a result, these approaches can lead to redundant or inefficient deployments. This paper addresses this gap by first determining the minimum number of required chargers using a graph-theoretic approach, followed by optimizing their positions using an enhanced metaheuristic method.

The main contributions of this work are summarized as follows:

- The purpose of this paper is to propose a novel hybrid framework that integrates the Degree of Saturation Algorithm (DSA) with an Enhanced Grey Wolf Algorithm (EGWA) for optimal charger placement in WSNs.
- Employing DSATUR to minimize the number of chargers through efficient sensor grouping, followed by EGWA to maximize coverage through optimal charger positioning.
- Introducing a local enhancement mechanism in EGWA to adaptively relocate idle chargers to uncovered sensors, thereby improving robustness across dense and sparse deployments.

To this end, here we propose a hybrid approach: first, the DSATUR algorithm is used to redundancy of the charger by grouping sensors so that each group can share one charger. Then, the EGWA is used to find the optimal positions for these chargers. The enhanced version improves the original Grey Wolf Algorithm (GWA) with local search techniques, leading to better convergence and performance in diverse deployment scenarios.

Limitation: In this study, we consider a *static wireless sensor network* where both sensors and chargers are assumed to have fixed positions once deployed. The focus is therefore on optimizing charger placement to ensure maximum coverage and efficient energy replenishment under static conditions. Dynamic or mobile network scenarios, where either sensors or chargers move over time, are beyond the scope of this work and are left for future research.

 Simulation results demonstrate that our proposed method significantly outperforms existing methods, including Haar (83%), Daubechies 2 (85%), Biorthogonal (86%), Symlets 8 (85%) wavelets, and the Raindrop (87%) and Blackhole (91%) algorithms. The proposed EGWA achieves the highest efficiency of 97%, indicating its robustness in real-world WRSNs.

 The rest of the paper is structured as follows: Section II presents the related work in wireless sensor recharging. Section III formulates the charger placement problem. Section IV details the Degree of Saturation implementation. Section V introduces the Grey Wolf Algorithm, and Section VI explains the enhancements proposed in Enhanced Grey Wolf Algorithm. Section VII discusses the simulation results and performance evaluation. Finally, Section VIII concludes the paper and outlines future directions.

## Related work

Placing wireless chargers to replenish energy in sensor networks has garnered significant research attention in recent years. Numerous studies have addressed related challenges, with many focusing on maximizing charging utility through various approaches and perspectives. Balaji and Arivudainambi^16^ proposed an approach using the Daubechies wavelet algorithm to optimize charger placement. This algorithm leverages the translation and dilation properties of Daubechies wavelets, allowing chargers to adjust both vertically and horizontally, as well as repositioning redundant chargers to more effective locations. The method was compared against four other wavelets–Haar, Daubechies 2 (db2), Biorthogonal 55 (bio55), and Symlets–and demonstrated superior performance. Notably, this approach also calculates the precise number of chargers needed, ensuring minimal redundancy.

Yang et al.^13^ proposed an improved Firefly algorithm to solve the charger deployment optimization problem and an optimization framework that simultaneously maximizes the coverage and the charging efficiency. Xingjian et al.^17^ addressed the optimal placement of wireless chargers by proposing two approximation algorithms tailored for omnidirectional charging: a greedy scheme and a relaxed rounding scheme. Their study provides performance guarantees for both algorithms, showing they can effectively determine charger placement to meet individual energy requirements in sensor networks. However, a method based solely on individual energy demands often results in a high number of chargers required to maintain network functionality.

Yu et al.^18^ tackled the connected wireless charger placement problem, where wireless chargers are able to communicate with each other. Their approach focuses on positioning a set number of wireless chargers within a network area to maximize overall charging utility, while ensuring that the chargers remain connected. This connectivity constraint adds a layer of complexity, aiming to optimize both coverage and communication between chargers. Hsin et al.^19^ proposed a charger deployment strategy using four metaheuristic algorithms: simulated annealing, tabu search, genetic algorithm, and ant colony optimization. To further improve performance, they introduced a layoff algorithm that enhances the effectiveness of these metaheuristic methods. Through comparative analysis, the study demonstrates the improvements achieved with LA across different algorithmic approaches.

Xue et al.^20^ introduced CHAIN (CHArging utIlity maximization), a pioneering approach that leverages wave interference to enhance wireless charging performance through optimal charger placement. CHAIN employs a two-step placement scheme: first, it determines initial charger positions to maximize the additive power from interfering waves; then, it fine-tunes each charger’s location to further increase the total charging utility across all sensors. This innovative method, which models and optimizes wave interference effects, is the first to use interference to boost overall charging efficiency in wireless sensor networks.

Haoran et al.^21^ proposed the RL-CCD (Reinforcement Learning-based Charging Cluster Center Determination) algorithm for Optimal Charging Placement. Unlike direct clustering methods like K-Means++, RL-CCD uses reinforcement learning to iteratively adjust charging cluster center locations, optimizing placement for better coverage and energy efficiency. This approach enables more flexible and effective charger placement compared to traditional clustering algorithms.

Mallikarjuna et al.^22^ introduced a scheme for energy replenishment in wireless sensor networks (WSN) that combines both static and mobile chargers, managed through a reinforcement learning approach. Additionally, the scheme includes a dual topology mechanism, enabling mobile chargers to adapt to dynamic environmental conditions, enhancing charging efficiency in variable WSN scenarios.

Neelagandan and Balaji^23^ proposed a Grundy coloring algorithm to determine the minimum number of chargers and used the black hole algorithm to find their optimal placement. However, their results provided limited improvements compared to the outcomes presented in this work.

Table 1 summarizes related work for comparison, highlighting their contributions and limitations. While these studies provide a strong theoretical foundation and valuable insights, none directly address the critical aspects of our problem. Although prior efforts focus on optimal charger placement, they overlook the issue of redundant chargers. By calculating the precise number of required chargers, redundancy can be eliminated. The proposed coloring algorithm effectively addresses this gap by systematically determining the optimal number of chargers, ensuring redundancy is avoided while guaranteeing adequate coverage for all sensors.

**Table 1:** Comparison between the existing studies mentioned in the related work.

Paper	Determining No. of Chargers	Algorithm/Technique Used	Reducing Redundancy	Remarks
^16^	No	Daubechies Wavelet Algorithm	Yes	Efficient charger placement using wavelets, calculates positions but does not adapt dynamically.
^13^	No	Improved Firefly Algorithm	No	Optimizes coverage and efficiency, but does not minimize charger number, leading to possible redundancy.
^17^	No	Greedy + Relaxed Rounding Schemes	No	Focuses on individual energy requirements, may require excess chargers.
^18^	No	Connected Charger Placement Algorithm	No	Ensures connectivity among chargers, but redundancy is not addressed.
^19^	No	Simulated Annealing, Tabu Search, GA, ACO (+ Layoff Algorithm)	No	Metaheuristic combination improves deployment efficiency; redundancy still not fully addressed.
^20^	No	CHAIN (Wave Interference-based Scheme)	No	Innovative use of wave interference to boost charging, lacks optimization-based placement.
^21^	No	RL-CCD (Reinforcement Learning-based Charging Cluster)	Yes	Flexible placement via reinforcement learning, partially considers redundancy.
^22^	No	Reinforcement Learning with Static + Mobile Chargers	No	Incorporates mobile and static chargers; redundancy reduction limited.
^23^	Yes	Grundy coloring algorithm	Yes	Results provided limited improvements compared to the outcomes presented in this work.
Ours	Yes	Hybrid Coloring + Optimization Algorithm	Yes	Determines minimum chargers, eliminates redundancy, ensures full sensor coverage.

## Problem formation

Let $S=\{S_1,S_2,S_3,\dots ,S_m\}$ be the set of *m* sensors in the region $A\times A$ to be monitored, and $C=\{C_1,C_2,C_3,\dots ,C_n\}$ be the set of *n* chargers available to recharge the *m* sensors, where $n<m$. This means that the number of available chargers is fewer than the number of sensors that need to be recharged. Each charger could be placed near an individual sensor when *n* number of chargers is equal to or greater than the number of sensors. However, since chargers are fewer than the sensors, have to identify the optimal positions for the *n* charger so that all sensors are efficiently covered. The goal is to identify the optimal position of the *n* charger in such a way that the deployed chargers can recharge all *m* sensors. Each sensor $S_i$ is located at position $(x_i,y_i)$ within the region, and each charger $C_j$ is positioned at $(x_j,y_j)$, with a charging range *r* that allows it to recharge the sensors within this range. A charger $C_j$ will recharge $S_i$ if it satisfies the following condition

1 $$\begin{aligned} (x_i-x_j)^2 + (y_i-y_j)^2\le r^2 \end{aligned}$$

The coverage of chargers can be represented in the form of a matrix,

2 $$\begin{aligned} \gamma =\begin{array}{*{20}c} C_{1} \\ C_{2} \\ \vdots \\ C_{n} \\ \end{array} \mathop {\left( {\begin{array}{*{20}c} & \alpha _{11} & \alpha _{12} & \dots & \alpha _{1m} \\ & \alpha _{21} & \alpha _{22} & \dots & \alpha _{2m} \\ & \vdots & \vdots & \ddots & \vdots \\ & \alpha _{n1} & \alpha _{n2} & \dots & \alpha _{nm} \\ \end{array}}\right) }\limits ^{\begin{array}{*{20}c} \quad S_1&\quad S_2&\dots&S_{m} \end{array}} \end{aligned}$$

where,

$$\alpha _{ij}={\left\{ \begin{array}{ll} 1, \text {if a sensor } S_j \text { monitor by Charger } C_i \\ 0, \text {otherwise}. \end{array}\right. }$$

The column sum of the $\gamma$ matrix is

$$\phi =[\phi _1,\phi _2,\dots ,\phi _i,\dots ,\phi _{m}]$$

where

$$\phi _i=\sum _{j=1}^{n} \alpha _{ij}$$

From the column sum $\phi$, the number of chargers covered the sensors can be calculated. The column sum $\phi$ is useful for identifying the sensors that are covered by more than one chargers at the same time, as well as for detecting redundant chargers. The total number of sensors recharged is referred to as the Quality of Coverage (QoC), and it is calculated using equation (3).

3 $$\begin{aligned} QoC=\sum _{j=1}^{m} \big \lceil \frac{\phi _i}{m} \big \rceil \end{aligned}$$

The percentage of *QoC* is used to quantify the proportion of sensors successfully covered by the deployed chargers. The percentage of *QoC* can be calculated by the equation given below:

4 $$\begin{aligned} Qoc[\%]=\frac{QoC}{m} \times 100 \end{aligned}$$

The QoC and its percentage are calculated using equations (3) and (4). This method enables the determination of QoC for any given region $A\times A$.

**Fig. 1:**
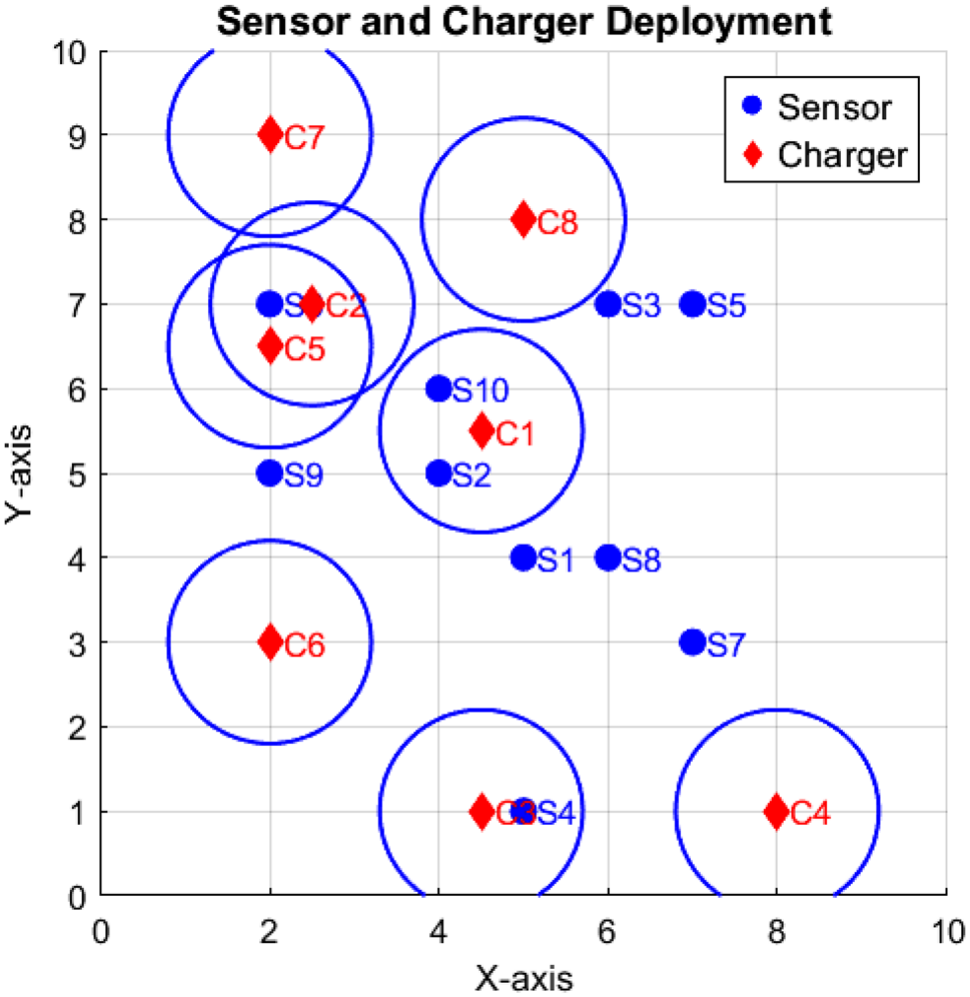
Sensors and chargers randomly deployed.

 Consider a sample network consisting of 10 sensors and 8 chargers with a charging range of 1 unit deployed randomly in the region $10\times 10$ as shown in Fig. 1. The matrix representation of charger coverage is identified using equation (1) and (2) is

$$\begin{aligned} \begin{array}{*{20}c} C_1\\ C_2\\ C_3 \\ C_4 \\ C_5 \\ C_6\\ C_7\\ C_8 \end{array} \mathop {\left( \begin{array}{*{20}c} & 0 & 1 & 0 & 0 & 0 & 0 & 0 & 0 & 0 & 1 \\ & 0 & 0 & 0 & 0 & 0 & 1 & 0 & 0 & 0 & 0\\ & 0 & 0 & 0 & 1 & 0 & 0 & 0 & 0 & 0 & 0\\ & 0 & 0 & 0 & 0 & 0 & 0 & 0 & 0 & 0 & 0\\ & 0 & 0 & 0 & 0 & 0 & 1 & 0 & 0 & 0 & 0\\ & 0 & 0 & 0 & 0 & 0 & 0 & 0 & 0 & 0 & 0\\ & 0 & 0 & 0 & 0 & 0 & 0 & 0 & 0 & 0 & 0\\ & 0 & 0 & 0 & 0 & 0 & 0 & 0 & 0 & 0 & 0\\ \end{array}\right) }\limits ^{\begin{array}{*{20}c}&S_1&S_2&S_3&S_4&S_5&S_6&S_7&S_8&S_9&S_{10} \end{array}} \end{aligned}$$

Thus the column sum of the matrix is $$\phi = \begin{pmatrix} 0 & 1 & 0 & 1 & 0 & 2 & 0 & 0 & 0 & 1\\ \end{pmatrix}$$

 The set of sensors recharged by the chargers are $S_1=\{\}, S_2=\{C_1\}, S_3=\{\}, S_4=\{C_3\}$, $S_5=\{\}$, $S_6=\{C_2,C_5\}$, $S_7=\{\}$, $S_8=\{\}$, $S_9=\{\}$ and $S_{10}=\{C_1\}$ respectively.

The Quality of coverage obtained using equations (3) and (4), $$QoC= \begin{pmatrix} 0 + 1 + 0 + 1 + 0 + 1 + 0 + 0+0+1\\ \end{pmatrix}$$ = 4 and the $Qoc[\%]$ = 40% and the total number of chargers used is 8. For a sample network with 10 sensors, identifying redundant chargers and finding optimal positions for efficient recharging is easily manageable. However, as the network grows larger, the complexity of this task increases. As a result, it becomes critical to calculate the charger requirements and to optimize their placement to minimize redundancy and prolong the network’s overall lifespan algorithm needed.

Existing literature on finding the optimal positions for chargers to recharge sensors includes various algorithms, such as mathematical-based algorithms^24^, evolutionary-based algorithms^12,13^, and so on. Despite numerous studies, the challenge of determining the minimum number of chargers required to recharge all sensors in a region remains largely unaddressed. In this paper, the Degree of Saturation method is proposed to calculate the minimum charger requirement needed to recharge the sensors.

**Algorithm 1:**
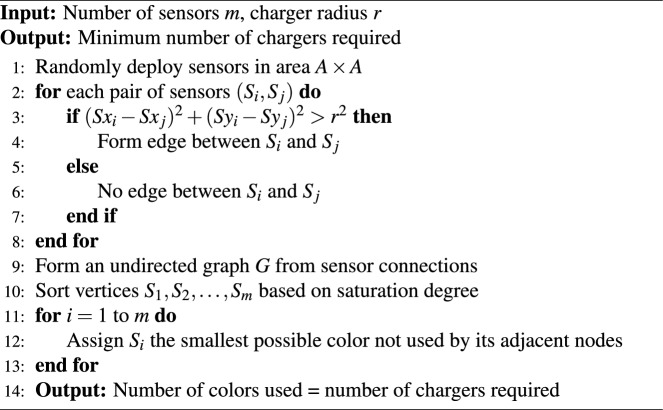
Implementing Degree of Saturation Algorithm

## Determining charger requirement using degree of saturation

### Degree of saturation

The number of chargers needed to recharge all sensors in a network is vital to minimizing costs and energy consumption. To determine this, the concept of vertex coloring can be effectively utilized. In this approach, other methods (Grundy and Welsh-Powell) assign color to vertices based on their vertex degree and associated heuristics, ensuring that adjacent vertices receive different colors. Each unique color corresponds to a distinct charger, providing an efficient estimate of the minimum charger count. The DSA prioritizes vertices based on their saturation degree, the number of distinct colors used by their neighbors. This approach helps prioritize vertices that are constrained, i.e., have neighbors already assigned multiple colors. Therefore, in this paper, the DSA is used for coloring the graph to identify charger requirements.

### Illustrative example: DSATUR on a WSN (Reproducible)

**Setup.** We consider six sensors with fixed coordinates (arbitrary units):

$$S_1=(0.0,0.0),\ S_2=(1.8,0.6),\ S_3=(3.6,0.2),\ S_4=(0.6,2.3),\ S_5=(3.1,2.3),\ S_6=(5.0,0.8).$$

We adopt the *sensor-anchored charger* model with charger range $r=2.5$. Two sensors cannot share a single charger if their Euclidean distance exceeds $r$. Accordingly, we form an undirected edge between $S_i$ and $S_j$ iff $\Vert S_i - S_j\Vert> r$.

**Conflict graph (edges).** Using the rule above, the edges are:

$$\{(S_1,S_3), (S_1,S_5), (S_1,S_6), (S_2,S_6), (S_3,S_4), (S_4,S_6)\}.$$

The corresponding vertex degrees are: $\deg (S_1)=3,\ \deg (S_6)=3,\ \deg (S_3)=2,\ \deg (S_4)=2,\ \deg (S_2)=1,\ \deg (S_5)=1.$

**DSATUR run.** At each step, choose the uncolored vertex with the largest *saturation degree* (number of distinct colors already used in its neighborhood); break ties by larger degree.

**Table 2:** Coloring process in DSATUR for a small sensor network.

Step	Chosen vertex	Neighbor colors	Assigned color	Colored set
1	$S_1$ (sat=0, deg=3)	–	$c_1$	$\{S_1\}$
2	$S_6$ (sat=1, deg=3)	$\{c_1\}$	$c_2$	$\{S_1,S_6\}$
3	$S_3$ (sat=1, deg=2)	$\{c_1\}$	$c_2$	$\{S_1,S_6,S_3\}$
4	$S_4$ (sat=1, deg=2)	$\{c_2\}$	$c_1$	$\{S_1,S_6,S_3,S_4\}$
5	$S_2$ (sat=1, deg=1)	$\{c_2\}$	$c_1$	$\{S_1,S_6,S_3,S_4,S_2\}$
6	$S_5$ (sat=1, deg=1)	$\{c_1\}$	$c_2$	$\{S_1,S_6,S_3,S_4,S_2,S_5\}$

**Result.** DSATUR uses only two colors:

$$\mathcal {C}_1=\{S_1,S_4,S_2\},\qquad \mathcal {C}_2=\{S_6,S_3,S_5\}.$$

From Table 2, it is observed that the *minimum* number of colors required is 2, which directly corresponds to the minimum number of chargers needed for this instance under the proposed model. By extending this method, the required number of chargers can be systematically determined for larger and more complex sensor networks. The reason is that the WPA and GCA colors vertices based on the degree (number of neighbors), starting with the highest-degree vertex and continuing to the next vertices.

### DSA based coloring for sample network

 In the problem formulation section, a sample network was considered to illustrate the working of different graph coloring methods. The DSA provides better results compared to the Welsh-Powell Algorithm (WPA) and the Grundy Coloring Algorithm (GCA), as shown in Table 3. The key distinction lies in the coloring strategy: WPA and GCA assign colors to vertices primarily based on their degree (i.e., the number of neighbors), starting with the highest-degree vertex and proceeding sequentially. In contrast, DSA prioritizes vertices according to their saturation degree, which dynamically considers the number of distinct colors already assigned to adjacent vertices. This allows DSA to make more adaptive coloring decisions, thus reducing the total number of colors and, consequently, the number of chargers required.

**Table 3:** Comparison Between the Algorithms for Determining the Number of Chargers Needed.

Area	Sensors	Radius	Grundy	Welsh-Powell	DSA
100	100	5	78	76	75
250	200	10	145	143	141
500	300	15	219	217	216
750	400	20	290	287	285
1000	500	25	349	346	345

To determine the number of charger needed to recharge the sensors using DSA, the sensors are modeled as vertices, with adjacency defined by the condition given in equation (5).

5 $$\begin{aligned} (x_i-x_j)^2 + (y_i-y_j)^2>r^2 \end{aligned}$$

where $(x_i,y_i)$ and $(x_j,y_j)$ are the spatial coordinates of ${S_i}$ and ${S_j}$ sensors respectively. An edge is established between two sensors only if their distance exceeds the charger range *r*. If the distance is within *r*, no edge is formed.

The adjacent matrix of all sensors identified using equation (5) is

6 $$\begin{aligned} D = \begin{pmatrix} S_{11} & S_{12} & \dots & S_{1j} \\ S_{21} & S_{22} & \dots & S_{2j} \\ \vdots & \vdots & \ddots & \vdots \\ S_{i1} & S_{i2} & \dots & S_{ij} \\ \end{pmatrix} \end{aligned}$$

where,

$$S_{ij}={\left\{ \begin{array}{ll} 1, \text {if equation (<span class='crossLinkCiteEqu'>5</span>) satisfy} \\ 0, \text {otherwise}. \end{array}\right. }$$

$$S_{ij}, 1\le i,j \le m$$

The adjacency matrix *D* is constructed for the sample network given in Fig. 1 using equation (6).

$$D= \begin{pmatrix} 0 & 1 & 1 & 1 & 1 & 1 & 0 & 1 & 1 & 1\\ 1 & 0 & 1 & 1 & 1 & 1 & 1 & 1 & 1 & 0\\ 1 & 1 & 0 & 1 & 0 & 1 & 1 & 1& 1 & 1\\ 1 & 1 & 1 & 0 & 1 & 1 & 1 & 1& 1 & 1\\ 1 & 1 & 0 & 1 & 0 & 1 & 1 & 1& 1 & 1\\ 1 & 1 & 1 & 1 & 1 & 0 & 1 & 1& 1 & 1\\ 1 & 1 & 1 & 1 & 1 & 1 & 0 & 1& 1 & 1\\ 0 & 1 & 1 & 1 & 1 & 1 & 1 & 0& 1 & 1\\ 1 & 1 & 1 & 1 & 1 & 1 & 0 & 1& 0 & 1\\ 1 & 0 & 1 & 1 & 1 & 1 & 1 & 1 & 1 & 0\\ \end{pmatrix}$$

**Fig. 2:**
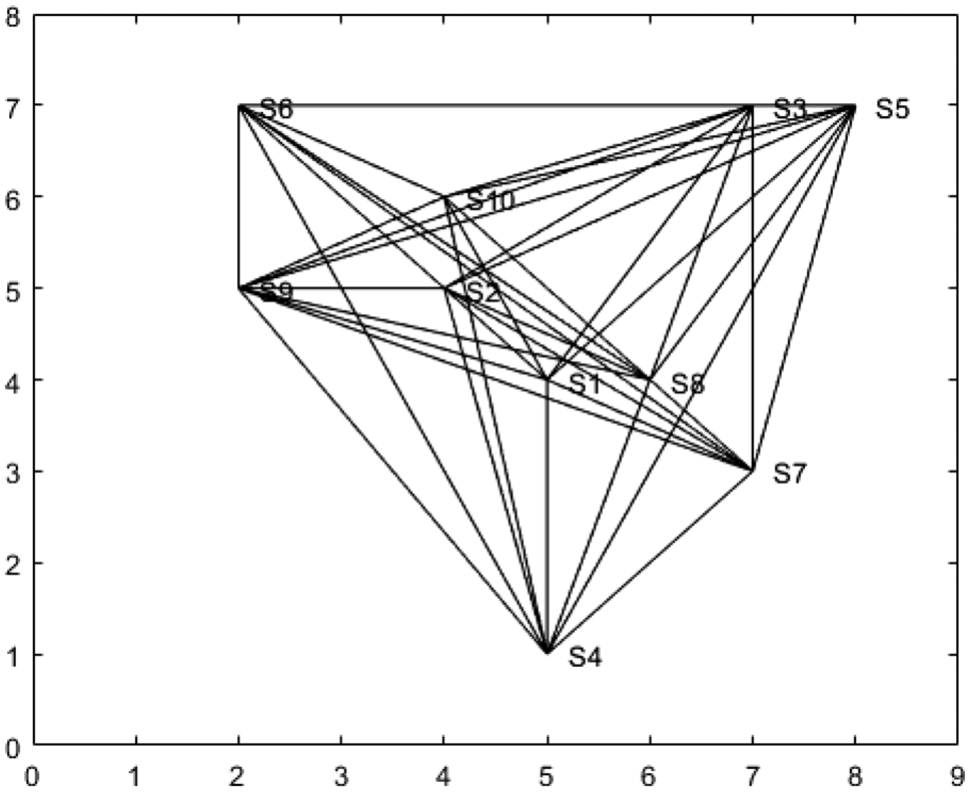
Graph construction for the sensors network.

A graph $G$ is constructed based on the adjacency matrix $D$, as shown in Fig. 2. The coloring process begins with the uncolored vertex $S_i$ that has the highest saturation degree. Initially, all vertices have a saturation degree of zero, so the algorithm starts with vertex $S_1$. Assign $S_1$ the color $k$, where $k$ is the smallest integer from the set $k = \{1, 2, \dots , n\}$, with each number representing a different color, and is not used by any of the neighbors of $S_1$. Once $S_1$ is colored, the vertex with the highest saturation degree is selected for the next coloring. If there are multiple vertices with the same saturation degree, the one with the highest degree (most neighbors) is chosen. Assign this vertex the smallest available color that has not been used by its neighbors. This process is repeated for the remaining uncolored vertices.

**Fig. 3:**
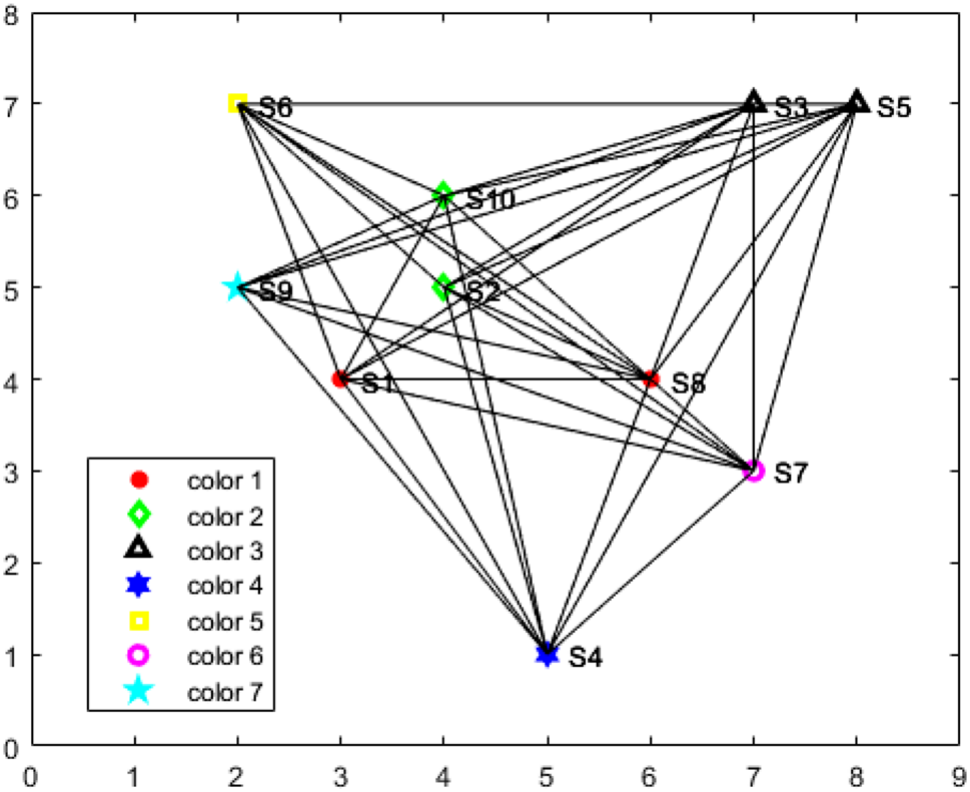
Vertex coloring using DSA.

After the vertices are colored using the DSA, the number of distinct colors in the set *k* corresponds to the required number of chargers, denoted *n*. The minimum number of colors needed for a given graph can be determined using Algorithm 1. Sensors assigned the same color are near and can share a single charger. In the sample network considered, the graph is colored using seven distinct colors, which implies that seven chargers are required.Thus, any redundant chargers can be eliminated. For instance, in the sample network, charger $C_8$ is removed, as illustrated in Fig. 3. The EGWA is then applied to determine the optimal placement of the remaining chargers, ensuring efficient recharging of all sensors.

## Grey Wolf algorithm

 The Grey Wolf Algorithm was first introduced by Seyedali Mirjalili in 2013^15^. The GWA is modeled after the social structure of grey wolves (Canis lupus), which are part of the Canidae family. Grey wolves live in packs governed by a strict dominance hierarchy. At the top are the alpha wolves, typically a dominant male and female, who make key decisions for the pack, such as determining when to hunt, where to rest, and when to wake. The rest of the pack follows their lead. These alpha wolves hold the highest authority, which is why they are also known as the dominant wolves, and only they are allowed to mate within the pack.

The beta wolves form the second tier of the hierarchy. They are subordinate to the alphas but play a crucial role in supporting them with decision-making and carrying out other duties within the pack. Betas can be either male or female, and they are the most likely to succeed the alpha if the current leader dies or becomes too old. However, betas must always remain loyal and obedient to the alphas.

The delta wolves rank below the alphas and betas. They are required to submit to the alphas and betas. This group includes Scouts, who monitor the territory’s borders and alert the pack to potential threats, Sentinels, responsible for protecting the pack, Elders, older wolves with experience, often former alphas or betas, Hunters, who provide food for the pack, and Caretakers, who look after the well-being of the other wolves.

At the bottom of the hierarchy are the omega wolves, who act as scapegoats. Omegas must submit to all the higher-ranking wolves and are the last to feed. The omega plays a key role in maintaining harmony within the pack, as the absence of an omega can lead to increased conflict among the other wolves. This hierarchical structure of leadership, coordination, and cooperation serves as the foundation for the GWA, simulating the behaviors observed in grey wolf packs.

As previously discussed, grey wolves surround their prey while hunting. To model this encircling behavior mathematically, the following equations have been introduced:

7 $$\begin{aligned} \vec {D}= & |\vec {C}.\vec {X}_p(t)-\vec {X}(t)| \end{aligned}$$

8 $$\begin{aligned} \vec {X}_k= & \vec {X}_p(t)-\vec {A}.\vec {D}, k=1,2,3 \end{aligned}$$

where *t* indicates the current iteration, $\vec {A}$ and $\vec {C}$ are coefficient vector, $\vec {X}_p$ is the position vector of the prey and $\vec {X}$ indicates the position vector of a grey wolf.

The vectors $\vec {A}$ and $\vec {C}$ are calculated as follows:

9 $$\begin{aligned} \vec {A}= & 2\vec {a}.\vec {r_1}-\vec {a} \end{aligned}$$

10 $$\begin{aligned} \vec {C}= & 2.\vec {r_2} \end{aligned}$$

where components of $\vec {a}$ are linearly decreased from 2 to 0 over the course of the iteration and $r_1$, $r_2$ are random vectors in [0,1].

**Fig. 4:**
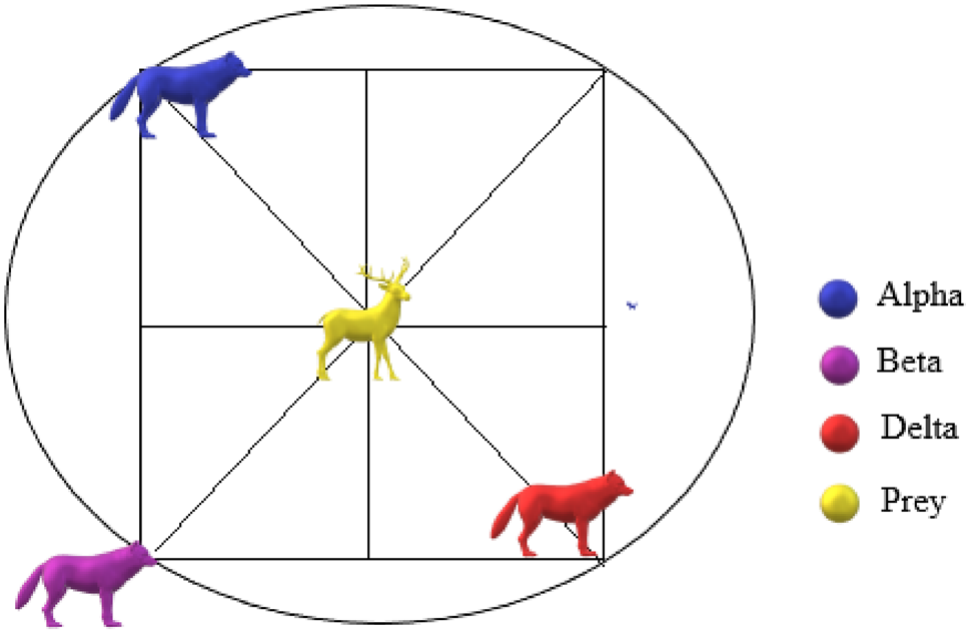
Hunting position of GWA.

 In Fig. 4, a grey wolf in the position of (*X*, *Y*) can update its position according to the position of the prey $(X', Y')$. Different places around the prey can be reached with respect to the current position by adjusting the value of $\vec {A}$ and $\vec {C}$ vectors. For instance, $(X'-X, Y)$ can be reached by setting $\vec {A}=(1,0)$ and $\vec {C}=(1,1)$. The random vectors $r_1$ and $r_2$ allow wolves to reach any position between the points in Fig. 4. So a grey wolf can update its position inside the space around the prey in any random location by using equations 7 and 8. The same concept can be applied to a search space with *n* dimensions, where the grey wolves navigate through hyper-cubes (or hyper-spheres) around the best solution found so far.

 Grey wolves possess the ability to detect the location of their prey and encircle them during a hunt, typically led by the alpha. Occasionally, the beta and delta wolves also participate in hunting. However, in an abstract search space, the exact location of the optimal solution (prey) is unknown. To simulate the hunting behavior of grey wolves mathematically, assume that the alpha (representing the best candidate solution), along with the beta and delta, have superior knowledge about the possible location of the prey. As a result, the three best solutions found so far are saved, and the other search agents (including omegas) update their positions based on the positions of these leading search agents. The following equations are proposed to model this process.

11 $$\begin{aligned} \vec {D}_{\alpha }= & |\vec {C}_1.\vec {X}_{\alpha }-\vec {X}|,\nonumber \\ \vec {D}_{\beta }= & |\vec {C}_2.\vec {X}_{\beta }-\vec {X}|,\nonumber \\ \vec {D}_{\delta }= & |\vec {C}_3.\vec {X}_{\delta }-\vec {X}| \end{aligned}$$

12 $$\begin{aligned} \vec {X}_1= & \vec {X}_{\alpha }-\vec {A}_1. (\vec {D}_{\alpha }),\nonumber \\ \vec {X}_2= & \vec {X}_{\beta }-\vec {A}_2.(\vec {D}_{\beta }),\nonumber \\ \vec {X}_3= & \vec {X}_{\delta }-\vec {A}_3.(\vec {D}_{\delta }) \end{aligned}$$

13 $$\begin{aligned} \vec {X}_{(t+1)}= & \frac{\vec {X}_1+\vec {X}_2+\vec {X}_3}{3} \end{aligned}$$

This describes how a search agent updates its position based on the positions of the alpha, beta, and delta in a 2D search space. The final position of the agent will be randomly located within a circle determined by the positions of the alpha, beta, and delta. In essence, the alpha, beta, and delta estimate the prey’s location, while the other wolves adjust their positions around the prey. This encircling behavior allows search agents to explore the space in a manner that reflects the wolves’ hunting strategy.

## Implementation of Enhanced Grey Wolf algorithm

 The GWA outperforms other evolutionary algorithms such as particle swarm optimization, gravity search algorithm, differential evolution, evolution programming, and evolution strategy^15^. Due to its superior performance, the GWA has been adapted and modified to determine the optimal placement of the chargers. In this paper, local enhancement techniques were applied to further refine the efficiency of GWA in identifying the best charger positions. A convergence comparison between GWA and EGWA is presented in Table 4, demonstrating the improved convergence behavior of the enhanced version. The step-by-step procedure for this optimization is detailed in Algorithm 2. In this approach, sensors are considered prey, while wolves represent chargers in the optimization process.

**Table 4:** Convergence Comparison between GWA and EGWA (5 independent runs).

Algorithm	Avg. Best Fitness	Std. Dev.	Avg. Iterations to Converge
GWA	0.684	0.012	145
EGWA	**0.721**	**0.007**	**93**

### Initial population

The number of population *p* was randomly generated with coordinates *n* that represent the position of the available chargers, and the chargers were placed based on the coordinates of the population. The size of the population is $2\times n$,

$$\begin{aligned} pop_p = \begin{pmatrix} x_1 & x_2 & \dots & x_n\\ y_1 & y_2 & \dots & y_n \end{pmatrix} \end{aligned}$$

For the sample network considered in problem formation, 7 chargers are needed to recharge all the sensors which is identified by DSA, and five populations with seven coordinates were randomly generated.

$$\begin{aligned} pop_1= & \begin{pmatrix} 2 & 7 & 4 & 2 & 1 & 5 & 0\\ 7 & 8 & 0 & 10 & 9 & 10 & 4 \end{pmatrix}\\ pop_2= & \begin{pmatrix} 1 & 0 & 8 & 0 & 2 & 4 & 2\\ 10 & 8 & 9 & 4 & 8 & 10 & 2 \end{pmatrix}\\ pop_3= & \begin{pmatrix} 7 & 5 & 6 & 2 & 4 & 2 & 6\\ 4 & 2 & 10 & 1 & 4 & 7 & 5 \end{pmatrix}\\ pop_4= & \begin{pmatrix} 4 & 2 & 3 & 8 & 1 & 6 & 7\\ 9 & 6 & 1 & 9 & 1 & 0 & 1 \end{pmatrix}\\ pop_5= & \begin{pmatrix} 2 & 8 & 7 & 1 & 4 & 6 & 2\\ 4 & 4 & 2 & 6 & 3 & 1 & 2 \end{pmatrix} \end{aligned}$$

**Algorithm 2:**
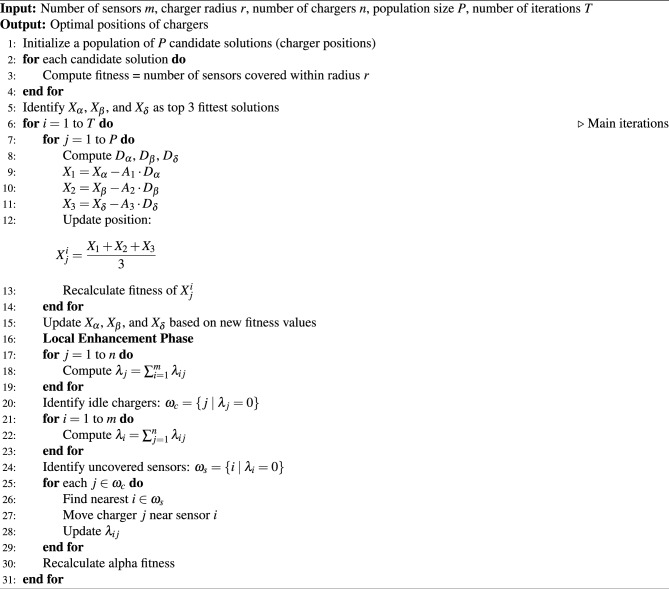
Implementing Enhanced Grey Wolf Algorithm (EGWA)

### Fitness value

 The fitness value is calculated for the initial population using equation (3), it represents the number of sensors recharged by each population. The fitness value for the considered five-sample population is

$$\begin{aligned} \begin{pmatrix} 5&4&1&1&2 \end{pmatrix} \end{aligned}$$

The solution with the highest fitness value was designated as Alpha, the second highest was named Beta and the third highest was named Delta. From the five-sample popultion, $pop_1$ is Alpha, $pop_2$ is Beta, and $pop_5$ is Delta based on fitness values. This hierarchical classification guided the optimization process.

### Population update

The Alpha, Beta, and Delta are chosen based on the fitness value.

$$\begin{aligned} Alpha= & \begin{pmatrix} 2 & 7 & 4 & 2 & 1 & 5 & 0\\ 7 & 8 & 0 & 10 & 9 & 10 & 4 \end{pmatrix}\\ Beta= & \begin{pmatrix} 1 & 0 & 8 & 0 & 2 & 4 & 2\\ 10 & 8 & 9 & 4 & 8 & 10 & 2 \end{pmatrix}\\ Delta= & \begin{pmatrix} 1 & 9 & 6 & 9 & 3 & 4 & 2\\ 1 & 6 & 1 & 6 & 5 & 0 & 1 \end{pmatrix} \end{aligned}$$

Equations (12) and (13) are used to update the position of the search agents, and a new position is determined.

$$\begin{aligned} D_{\alpha }= & (C_1*Alpha-pop_{1})\\ D_{\alpha }= & 0.26 \begin{pmatrix} 2 & 7 & 4 & 2 & 1 & 5 & 0\\ 7 & 8 & 0 & 10 & 9 & 10 & 4 \end{pmatrix}- \begin{pmatrix} 2 & 7 & 4 & 2 & 1 & 5 & 0\\ 7 & 8 & 0 & 10 & 9 & 10 & 4 \end{pmatrix}\\ D_{\alpha }= & \begin{pmatrix} 0.22 & 1.98 & 1.32 & 1.98 & 0.66 & 0.88 & 0.44\\ 0.22 & 1.32 & 0.22 & 1.32 & 1.10 & 0 & 0.22 \end{pmatrix} \end{aligned}$$

Similarly, ${D}_{\beta }$ and ${D}_{\delta }$ were calculated

$$\begin{aligned} {D}_{\beta }= & \begin{pmatrix} 1.64 & 2.34 & 3.29 & 4.42 & 6.45 & 2.34 & 9.47\\ 6.39 & 1.42 & 7.89 & 6.15 & 5.70 & 5.73 & 1.17 \end{pmatrix}\\ {D}_{\delta }= & \begin{pmatrix} 6.15 & 8.55 & 6.84 & 4.56 & 3.17 & 4.02 & 4.56\\ 6.41 & 6.14 & 5.21 & 4.61 & 2.51 & 4.54 & 2.98 \end{pmatrix}\\ X_1= & Alpha-A_1*{D}_\alpha \\ X_1= & \begin{pmatrix} 2 & 7 & 4 & 2 & 1 & 5 & 0\\ 7 & 8 & 0 & 10 & 9 & 10 & 4 \end{pmatrix} -0 *\begin{pmatrix} 0.22 & 1.98 & 1.32 & 1.98 & 0.66 & 0.88 & 0.44\\ 0.22 & 1.32 & 0.22 & 1.32 & 1.10 & 0 & 0.22 \end{pmatrix}\\ X_1= & \begin{pmatrix} 2 & 7 & 4 & 2 & 1 & 5 & 0\\ 7 & 8 & 0 & 10 & 9 & 10 & 4 \end{pmatrix}\\ \end{aligned}$$

Similarly, $X_2$ and $X_3$ value calculated

$$\begin{aligned} X_2= & \begin{pmatrix} 4.24 & 5.84 & 6.90 & 8.49 & 9.55 & 5.84 & 6.37\\ 8.49 & 9.02 & 8.49 & 2.65 & 6.90 & 7.43 & 6.37 \end{pmatrix}\\ X_3= & \begin{pmatrix} 2.65 & 3.65 & 4.31 & 5.31 & 5.97 & 3.65 & 3.98\\ 5.31 & 5.64 & 5.31 & 1.65 & 4.31 & 4.64 & 3.98 \end{pmatrix} \end{aligned}$$

Using Equation (14) new positions were calculated

$$\begin{aligned} {X}^1_1= & \frac{{X}_1+{X}_2+{X}_3}{3}\\ X^1_1= & \begin{pmatrix} 3.71 & 5.11 & 6.04 & 7.43 & 8.36 & 5.11 & 5.57\\ 7.43 & 7.89 & 7.43 & 2.32 & 6.04 & 6.50 & 5.57\\ \end{pmatrix} \end{aligned}$$

The fitness value computed for the updated population is 6 and the updated population is compared with the previous one. Based on fitness values, the best population is selected for further processing. This similar process is performed to update the remaining population. The fitness value of the five updated populations is

$$\begin{aligned} \text {fitness value=} \begin{pmatrix} 6&4&2&3&5 \end{pmatrix} \end{aligned}$$

In this approach, new Alpha, Beta, and Delta candidates are determined. However, there is a possibility that the fitness values do not exhibit significant improvement across iterations. To address this issue, a local enhancement method is introduced to refine fitness value, ensuring better convergence and optimization performance.

### Local enhancement phase

 The local enhancement step is highly beneficial in enhancing fitness values. The main idea is to identify chargers that are *idle* (i.e., not recharging any sensor) and reposition them to cover uncovered sensors, thereby improving the overall solution quality. By computing the coverage matrix using Equation (15), idle chargers can be systematically detected and used to enhance the result. This refinement not only optimizes charger placement but also improves overall efficiency, ensuring a higher fitness value with minimal iteration.

#### Step 1: Constructing the coverage matrix

The *coverage matrix* $\lambda$ is formed for the best population ($X^1_1$). It defines as follows:

14 $$\begin{aligned} \lambda = \begin{pmatrix} \lambda _{11} & \lambda _{12} & \dots & \lambda _{1n} \\ \lambda _{21} & \lambda _{22} & \dots & \lambda _{2n} \\ \vdots & \vdots & \ddots & \vdots \\ \lambda _{m1} & \lambda _{m2} & \dots & \lambda _{mn} \\ \end{pmatrix} \end{aligned}$$

where

$$\lambda _{ij} = {\left\{ \begin{array}{ll} 1, & \text {if sensor } S_i \text { is covered by charger } C_j, \\ 0, & \text {otherwise}. \end{array}\right. }$$

This binary matrix provides a complete mapping between chargers and the sensors they cover. The coverage matrix for $X^1_1$ is shown below:

$$\begin{aligned} \lambda = \begin{array}{*{20}c} S_1 \\ S_2 \\ S_3 \\ S_4 \\ S_5 \\ S_6 \\ S_7 \\ S_8 \\ S_9 \\ S_{10} \\ \end{array} \mathop {\left( \begin{array}{*{20}c} & 0 & 0 & 0 & 0 & 0 & 0 & 1 \\ & 0 & 0 & 0 & 0 & 0 & 0 & 0 \\ & 0 & 1 & 0 & 0 & 0 & 0 & 0 \\ & 0 & 0 & 1 & 0 & 0 & 0 & 0 \\ & 1 & 0 & 0 & 0 & 0 & 0 & 0 \\ & 0 & 0 & 0 & 0 & 1 & 0 & 0 \\ & 0 & 0 & 0 & 0 & 0 & 0 & 0 \\ & 0 & 0 & 0 & 0 & 0 & 0 & 0 \\ & 0 & 0 & 0 & 0 & 0 & 0 & 1 \\ & 0 & 0 & 0 & 0 & 0 & 0 & 0 \\ \end{array}\right) }\limits ^{\begin{array}{*{20}c}&C_1&C_2&C_3&C_4&C_5&C_6&C_7 \end{array}} \end{aligned}$$

#### Step 2: Identifying idle chargers

The total coverage contribution of charger $C_j$ is computed as:

15 $$\begin{aligned} \lambda _j = \sum _{i=1}^{m} \lambda _{ij}, \quad j=1,2,\dots ,n \end{aligned}$$

If $\lambda _j=0$, then the charger $C_j$ does not charge any sensor and is therefore idle. Idle chargers are formally identified as:

16 $$\begin{aligned} \omega _c = \Big \{ j \; \Big | \; \lambda _j = 0 \Big \} \end{aligned}$$

For the above example matrix ($\lambda$), $\omega _c = \{4,6\}$, which means that the chargers $C_4$ and $C_6$ are idle.

#### Step 3: Identifying uncovered sensors

Similarly, the number of chargers that cover each sensor $S_i$ is identified by:

17 $$\begin{aligned} \lambda _i = \sum _{j=1}^{n} \lambda _{ij}, \quad i=1,2,\dots ,m \end{aligned}$$

If $\lambda _i=0$, then the sensor $S_i$ is not covered by any charger. The set of uncovered sensors is therefore:

18 $$\begin{aligned} \omega _s = \Big \{ i \; \Big | \; \lambda _i = 0 \Big \} \end{aligned}$$

From the example, $\omega _s = \{2,7,8,10\}$, corresponding to the sensors $S_2, S_7, S_8,$ and $S_{10}$.

#### Step 4: Repositioning idle chargers

The idle chargers in $\omega _c$ are repositioned near the uncovered sensors in $\omega _s$. For example, placing chargers $C_4$ and $C_6$ near $S_2$ and $S_7$ increases the total number of covered sensors from 6 to 8, thus improving the fitness value.

This process is repeated iteratively, and the updated charger positions are adopted as the new alpha solution in subsequent EGWA iterations. Eventually, in the $n^{th}$ iteration, all sensors are covered and the fitness value reaches its maximum.

This step-by-step refinement ensures that the enhanced algorithm can dynamically reposition chargers, improving both coverage and convergence speed.

The updated charger positions with improved fitness are taken as the new alpha and carried forward to the next iteration. This process guarantees a gradual improvement in the fitness value with each iteration.

**Fig. 5:**
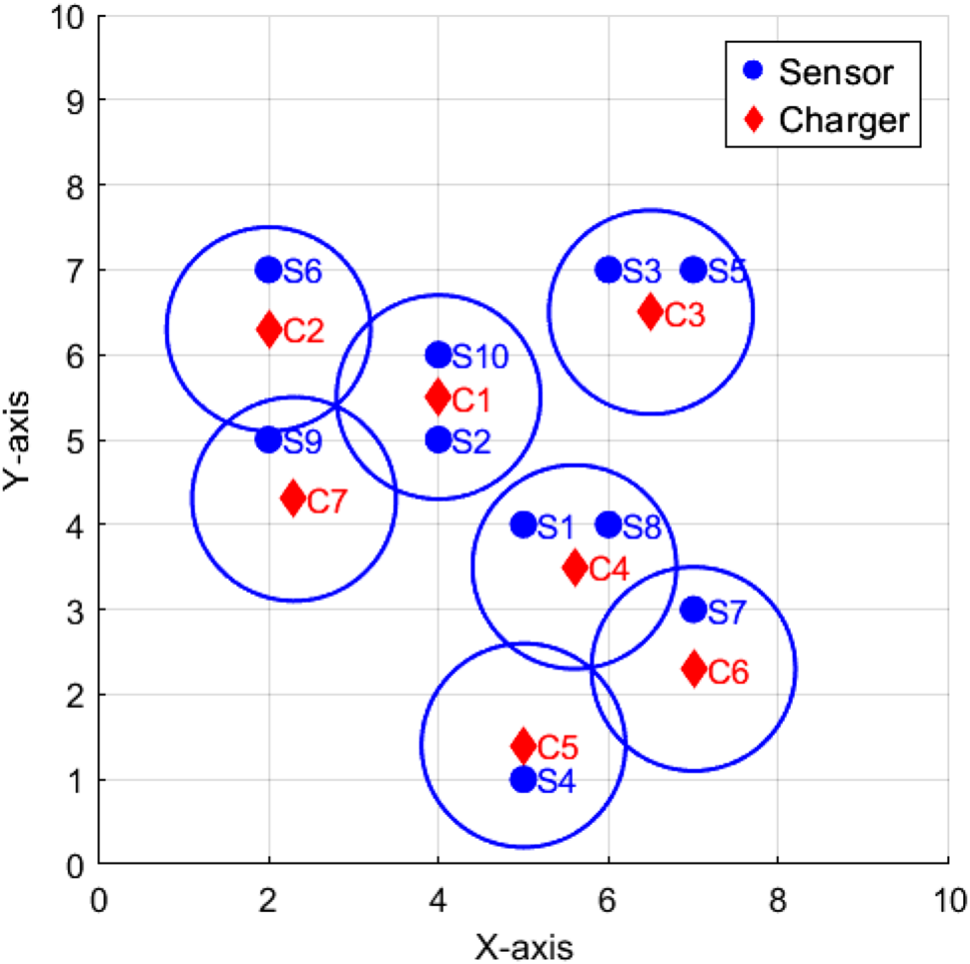
Optimal position of chargers using EGWA.

In the $n^{th}$ iteration the charger covered all the sensors as shown in Fig. 5. The fitness value is 10 and the best position is

$$\begin{aligned} \begin{pmatrix} 4 & 2 & 7.5 & 5.6 & 5 & 7 & 2.3\\ 5.5 & 6.3 & 6.5 & 3.5 & 1.4 & 2.3 & 4.3\\ \end{pmatrix} \end{aligned}$$

This result demonstrates the effectiveness of the optimization process, confirming that the enhanced algorithm is capable of dynamically refining charger placement to achieve full sensor coverage with a minimal number of iterations.

### Time complexity analysis

 Time complexity refers to the computational time an algorithm requires to complete its task as a function of the input size. It provides a theoretical measure of efficiency, particularly as the problem size increases.

 The EGWA is a population-based metaheuristic inspired by the hunting behavior of grey wolves. Its computational complexity is determined by the number of search agents (*P*), the number of iterations (*t*), the number of chargers (*n*), and the number of sensors (*m*). The overall time complexity analysis is summarized below.

**Best Case:** Occurs when EGWA converges early due to favorable initial positions, requiring only $t_{best}$ iterations:

$$\begin{aligned} T_{best} = O(t_{best} \cdot P \cdot n \cdot m) \end{aligned}$$

**Worst Case:** Occurs when EGWA requires the maximum allowed iterations *t*, with full updates and fitness evaluations:

$$\begin{aligned} T_{worst} = O(t \cdot P \cdot n \cdot m) \end{aligned}$$

**Average Case:** Typically, convergence occurs in $t_{avg}$ iterations, reflecting realistic problem behavior:

$$\begin{aligned} T_{avg} = O(t_{avg} \cdot P \cdot n \cdot m) \end{aligned}$$

In all cases, the dominant factor is fitness evaluation, which requires computing charger–sensor coverage relationships ($O(n \cdot m)$ per agent). Thus, EGWA has an overall complexity of $O(t \cdot P \cdot n \cdot m)$. Although slightly higher than the standard Grey Wolf Optimizer due to the *local enhancement phase*, the added cost is linear and does not alter the asymptotic behavior.

**Practical Justification:**

- The additional cost of local enhancement is negligible compared to the savings achieved in reducing the number of deployed chargers and ensuring full coverage.
- In real-world WSNs, sensor nodes are relatively static and its position are corrected in the backspace; therefore, EGWA’s complexity remains acceptable for practical deployment.
- Simulation results (Section 7) show that EGWA converges within a limited number of iterations ($t_{avg} \ll t$), keeping the runtime within feasible limits even for large-scale networks.
- Compared with other advanced metaheuristics, EGWA achieves superior coverage and charger utilization with quite higher execution time in practice.

Hence, the slightly higher complexity is justified by the significant improvements in solution quality, coverage efficiency, and robustness, making EGWA practical for real-time or near real-time wireless sensor network applications.

## Simulation result

 Simulation results were conducted in MATLAB to validate the performance of the proposed algorithm. Several studies have focused on wavelet-based methods for recharging sensors^25^ and enhancing network coverage^26^^27^. In this paper, the wavelet-based method Haar, Daubechies 2, biorthogonal, and Symlets 8 wavelets and evolutionary algorithm-based methods the raindrop algorithm and Black Hole Algorithm (BHA) were used for comparison to evaluate whether the EGWA provided the best results among all. The position of the sensor plays a major role in obtaining the QOC, therefore the position of the sensors is fixed for all the methods compared in this paper. The DSA determines the minimum number of chargers required for efficiently recharging sensors within a given region. The number of sensors ranged from 50 to 550, and the charging range varied between 10 to 150 in both the smaller $(100 \times 100)$ and the larger $(1000 \times 1000)$ regions. The iteration limit was set to 100, and the population limit was also set to 100 for all simulations. The results are reported as the mean coverage efficiency over 10 independent runs. The primary objective was to recharge all sensors using static wireless chargers determining their optimal positions.

**1. Performance Analysis of the proposed algorithm with other methods by varying a single parameter**

 In this section, sensor counts, charger range, and area are varied separately in region size $100 \times 100$ and $1000 \times 1000$. The iteration limit was set to 100, and the population limit was also set to 100 for all simulations. Comparative analysis of the results using different wavelets and evolutionary algorithms is discussed.

**Scenario 1.1**

In Table 5, the number of sensors ranges from 50 to 100, DSA identifies the minimum number of chargers needed, and the charger range is fixed at 10 units within a smaller ($100\times 100$) region, EGWA achieves up to 13% higher sensor coverage compared to wavelets, 10% higher than the raindrop algorithm, 8% higher than EGWA and 6% higher than BHA. EGWA consistently outperforms the other methods in coverage, with none of the other algorithms exceeding the 90% coverage of the recharge sensors. This suggests that EGWA is more effective in ensuring that a greater proportion of sensors are recharged within the specified constraints.

**Scenario 1.2**

**Fig. 6:**
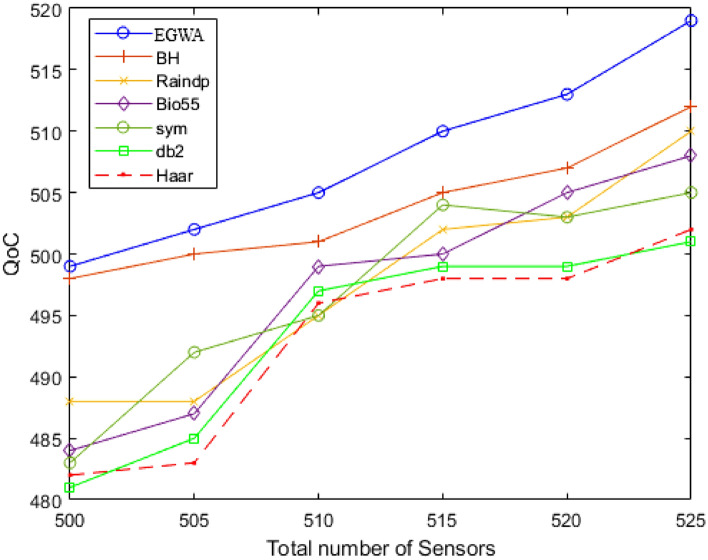
Evaluation of Results across Sensor Variations in Larger Area.

In this scenario, the number of sensors ranges from 500 to 525, DSA identifies the minimum number of chargers needed with the charger range fixed at 100 units across all variations in a larger ($1000\times 1000$) region, among the compared methods: wavelets, raindrop algorithm, and BHA. EGWA consistently achieves 99% sensor recharging coverage across all variations. In contrast, the other methods achieve coverage between 95% and 98%, highlighting EGWA’s superior performance in ensuring comprehensive sensor recharging. This consistent high performance underscores EGWA’s effectiveness in the given context. The QoC values for wavelets, the raindrop algorithm, BHA, and EGWA are illustrated in Fig. 6.

**Scenario 1.3**

**Fig. 7:**
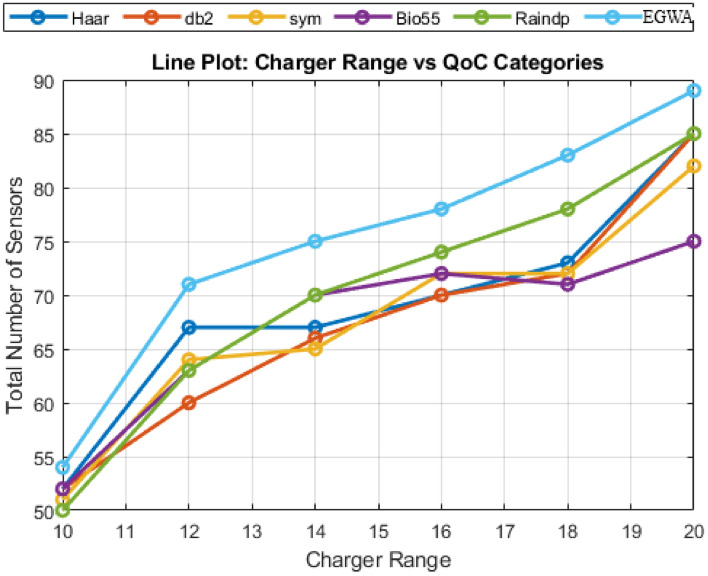
Evaluation of Results across Charger Range Variations in Smaller Region.

In this scenario, the charger range varies from 10 to 20 units, increasing by 2 units per variation, while the number of sensors is kept constant at 100, the charger needed is achieved by DSA and the sensor’s coordinates are constant for every variation within a smaller ($100\times 100$) region. Although neither the wavelets, raindrop algorithm, BHA, and EGWA reach the maximum sensor coverage in all of the variations, demonstrating its superior recharging efficiency. The performance of BHA stands out, especially given that other methods fall short of reaching the same level of effectiveness. The QoC values for wavelets, the raindrop algorithm, BHA, and EGWA are illustrated in Fig. 7, providing a clear comparison of their performance.

**Scenario 1.4**

 In Table 6, the charger range varies from 100 to 150 units, increasing by 10 units per variation, DSA identified the minimum number of chargers needed while the number of sensors remains constant at 100 within a larger ($1000\times 1000$) unit region. All compared methods, including wavelets and other algorithms–achieve the maximum level of sensor recharging at specific ranges. However, none of them achieves 100% coverage except EGWA, demonstrating superior performance across all variations. Notably, EGWA reaches this optimal level of performance earlier than the other methods, showcasing its effectiveness in achieving full sensor recharging with fewer adjustments.

**Scenario 1.5**

**Fig. 8:**
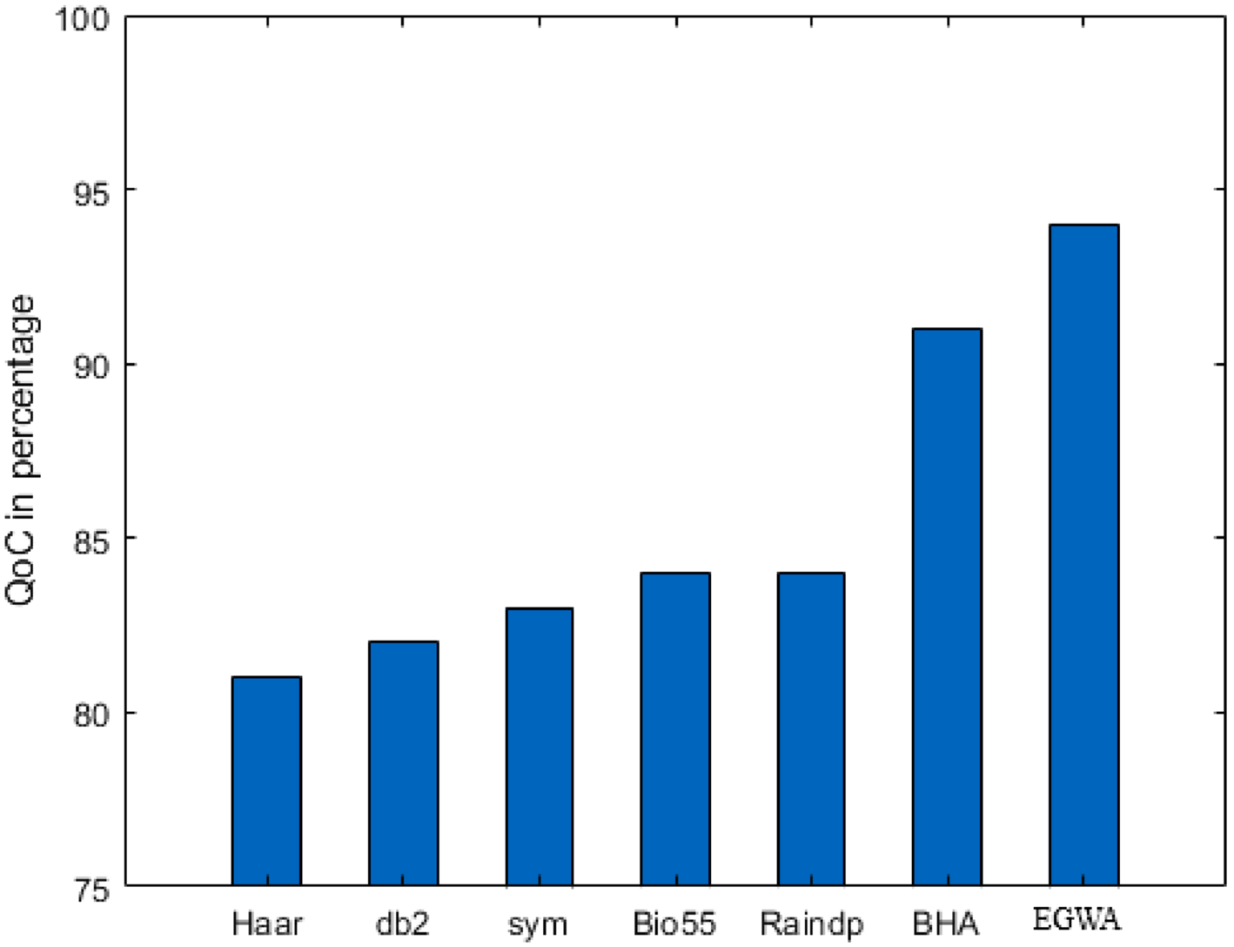
Results comparison for varying area.

In this scenario, the area of the region ranges from 50 to 300 units, DSA identifies the minimum number of chargers needed with the charger range set at 10% of the area and the number of sensors at 50% of the area. BHA consistently achieves the best performance as the area, sensor count, and charger range vary. Although the raindrop algorithm matches wavelets in some instances, EGWA exceeds all other methods in all variations and higher than GWA. Performance values, shown as percentages, indicate that EGWA is 2% more effective than the other methods. The QoC values for wavelets, the raindrop algorithm, BHA, and EGWA are illustrated in Fig. 8.

**Scenario 1.6**

 In Table 7, the region area ranges from 500 to 2500 units, DSA identifies the minimum number of chargers needed with the charger range fixed at 10% of the area and the number of sensors set at 50% of the area. This represents the largest area considered among the scenarios. Performance results indicate that the effectiveness of random deployment strategies diminishes in this larger context. EGWA consistently demonstrates superior performance compared to all other methods in this setting.

**2.Performance Analysis of the proposed algorithm with other methods by varying two parameters**

 In this section, significant variations are observed in the relationships between charger range vs sensor count, sensor count vs area, as well as area vs charger range. The iteration limit was set to 100, and the population limit was also set to 100 for all simulations. In addition, a comparative analysis of the results is conducted using different wavelets, and evolutionary algorithms are discussed.

**Scenario 2.1**

 In Table 8, the sensor count and charger range vary from 50-100 and 5-10 with DSA identifying the minimum number of chargers needed, and region fixed at $100\times 100$. This represents the largest area considered among the scenarios. Performance results indicate that the effectiveness of random deployment strategies diminishes in this larger context. EGWA consistently demonstrates superior performance compared to all other methods in this setting.

**Scenario 2.2**

**Fig. 9:**
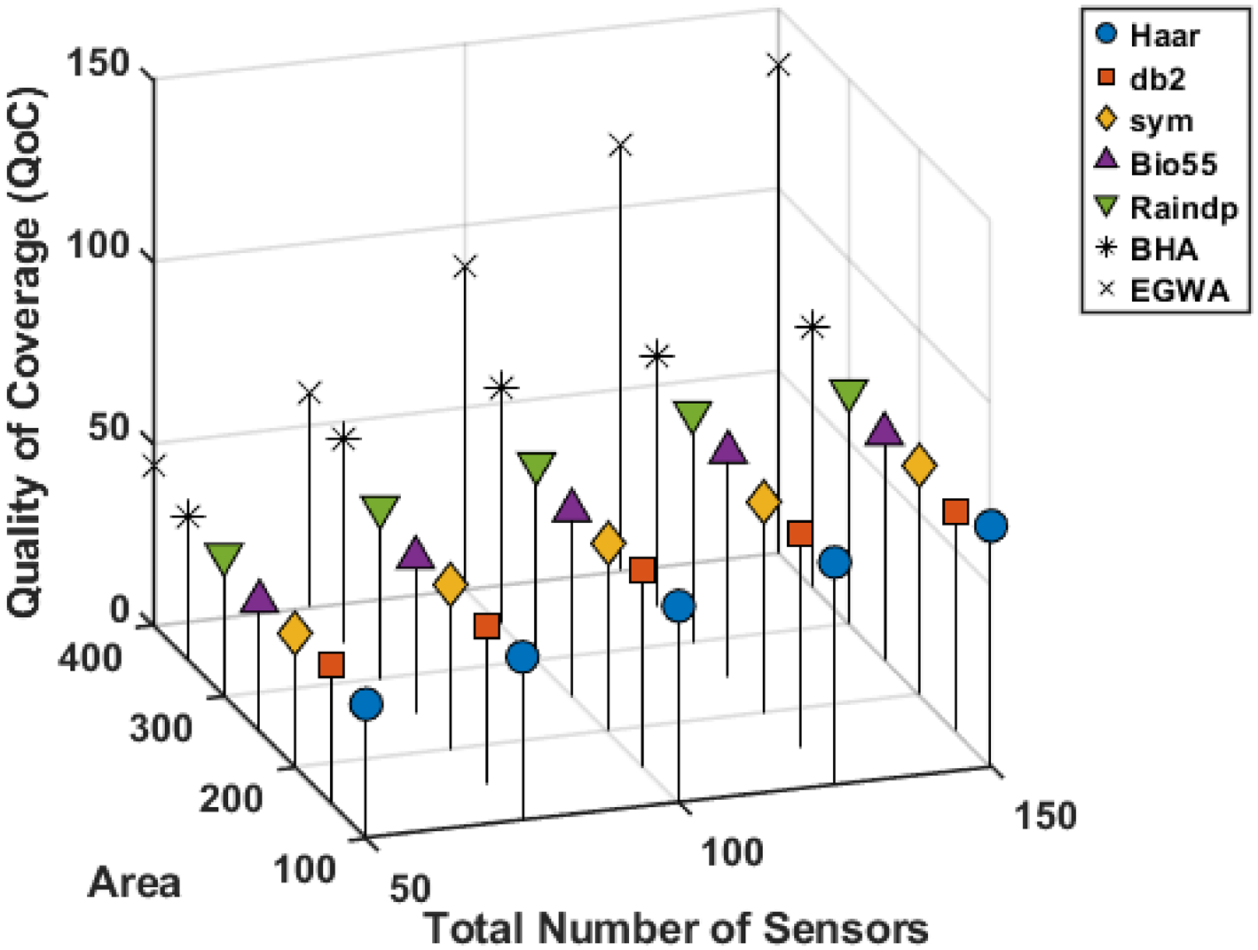
Results comparison for varying two parameters sensor and area.

In this scenario, the sensor count varies between 50 and 150, while the area ranges from 100 to 300. The DSA is utilized to determine the minimum number of chargers required, with the charger range fixed at 10 units. The results, as presented in Fig. 9, indicate that other algorithms failed to cover more than 80 sensors, while the EGWA successfully covered 136 of the total of 150 sensors, demonstrating its superior performance. This significant improvement highlights the effectiveness of EGWA in optimizing charger placement, ensuring efficient energy replenishment, and enhancing the overall sustainability of the sensor network.

**Scenario 2.3**

**Fig. 10:**
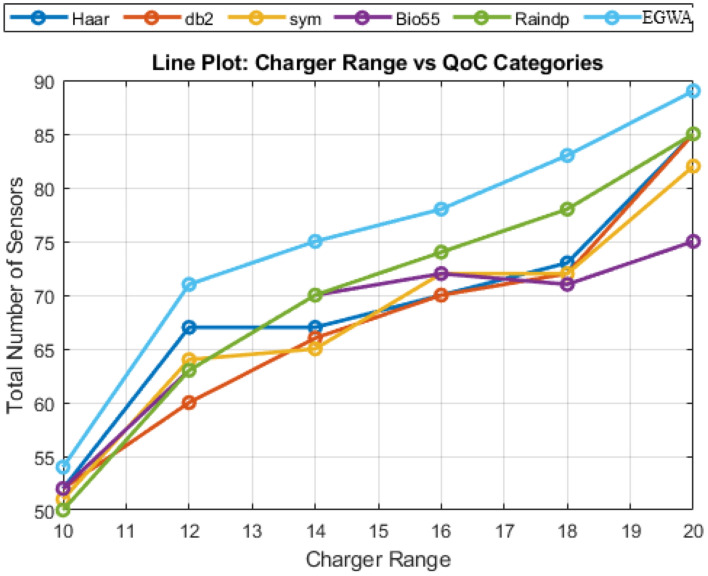
Results comparison for varying two parameters area and charger range.

In this scenario, the charging range varies between 10 and 30 units, while the area spans 100 to 300 units. The DSA is used to determine the minimum number of chargers required, with the number of sensors fixed at 100. The results, as presented in Fig. 10, reveal that other algorithms struggled to provide coverage beyond 90 sensors, leading to incomplete charging and potential inefficiencies in sensor network operations. In contrast, EGWA demonstrated superior performance by achieving nearly full sensor coverage, ensuring a more effective and reliable energy replenishment strategy. These findings underscore the efficiency of EGWA in optimizing charger placement and highlight its potential to improve the longevity and functionality of wireless sensor networks.

**3.Performance Analysis of the proposed algorithm with other methods by varying three parameters**

Table 9 presents a comparative analysis of different algorithms (Random, Haar, db2, sym, bio55, Raindrop, BHA, and EGWA) across varying area sizes, sensor counts, and charging ranges. As the area and sensor count increase, the number of sensors successfully covered by each algorithm also rises. However, significant variations in performance are observed. The EGWA consistently outperforms other algorithms, achieving the highest sensor coverage across all scenarios. For instance, at an area of 250 units with 250 sensors and a charging range of 25, the EGWA algorithm successfully covered 246 sensors which more than GWA, whereas other algorithms, such as Haar and db2, covered only 212 and 214 sensors, respectively. The iteration limit was set to 100, and the population limit was also set to 100 for all simulations. In particular, BHA also shows strong performance but remains slightly behind EGWA in most cases. These results highlight the superior efficiency of EGWA in optimizing charger placement and maximizing sensor coverage, making it a promising approach for large-scale wireless sensor networks.

In all scenarios, modify only a limited number of variables. Observing the overall changes across the entire population, statistical analysis will provide valuable insights.

**Table 5:** Performance of Algorithms for changing sensors count in smaller region.

Sensor	DSA	Random	haar	db2	sym	bio55	raindrop	BHA	GWA	EGWA
50	30	29	37	35	34	35	39	44	42	46
60	34	36	43	43	47	46	44	54	53	56
70	36	43	58	58	59	57	60	60	59	68
80	39	55	63	63	63	62	65	69	68	73
90	40	77	78	80	78	80	77	80	78	87
100	40	80	79	86	84	90	83	87	86	92

**Table 6:** Performance of Algorithms for changing charger range in larger region.

Charging range	DSA	Random	haar	db2	sym	bio55	raindrop	BHA	GWA	EGWA
100	54	69	82	82	82	86	85	88	86	94
110	52	81	88	88	87	87	90	92	90	94
120	48	76	90	94	90	91	93	95	93	97
130	47	85	94	95	94	93	93	97	95	98
140	42	86	95	94	92	94	95	98	96	100
150	39	87	95	97	97	95	96	99	97	100

**Table 7:** Performance of Algorithms for changing area largely.

Area	DSA	Random	haar	db2	sym	bio55	raindrop	BHA	GWA	EGWA
500	72	222	236	227	236	234	234	239	236	242
1000	89	482	484	481	476	483	484	490	487	492
1500	104	726	734	731	735	737	738	741	739	743
2000	124	964	988	987	981	988	988	990	988	995
2500	153	1203	1243	1236	1228	1232	1237	1246	1242	1248

**Table 8:** Performance of Algorithms for changing area largely.

Sensor	Charger range	DSA	Random	haar	db2	sym	bio55	raindrop	BHA	GWA	EGWA
50	5	41	12	17	19	19	20	22	30	28	40
60	6	44	26	31	32	34	34	35	36	34	58
70	7	54	36	40	40	42	42	45	54	52	65
80	8	49	51	53	54	55	55	57	63	61	66
90	9	45	63	65	67	68	68	71	78	74	79
100	10	47	76	80	83	85	84	89	90	89	93

**Table 9:** Area, Sensors and charger range varying in each case.

Area	Sensor	Charging range	Random	haar	db2	sym	bio55	raindrop	BHA	GWA	EGWA
50	50	5	30	32	35	34	35	39	43	42	50
100	100	10	62	68	69	72	75	85	91	89	98
150	150	15	104	112	115	116	121	125	139	136	147
200	200	20	155	161	163	163	168	176	184	180	196
250	250	25	198	212	214	219	218	227	237	234	246

### Time execution

The execution time analysis across different algorithms (BHA, Raindrop, Wavelet-based methods, and EGWA) indicates that execution time generally scales with the number of sensors, reflecting the direct influence of computational workload on sensor network size. Among these, the Wavelet-based approach consistently achieved the lowest execution time, demonstrating its computational efficiency. The Raindrop method exhibited moderate execution times, balancing computational cost with acceptable solution quality. BHA required comparatively higher execution time due to its iterative, population-based search mechanism, but its reliability in identifying optimal charger placements justifies the additional cost.

EGWA recorded the highest execution time across all sensor counts. This increase is primarily due to the additional adaptive operations and diversified exploration strategies embedded in the enhanced algorithm. Despite the computational overhead, EGWA provides the best convergence accuracy and solution quality, particularly in complex and large-scale sensor deployment scenarios. Since the objective of this work emphasizes solution reliability and optimality over computational speed, the additional time cost of EGWA is considered acceptable, making it the most robust choice for precision-critical WSN charger placement problems.

## Statistical analysis using ANOVA and Tukey HSD

 Statistical analysis plays a pivotal role in algorithm comparison by offering objective, reproducible evidence of performance differences. It ensures that conclusions are not based on random variation but grounded in statistically significant outcomes. In this work, we employed both Analysis of Variance (ANOVA) and Tukey’s Honestly Significant Difference (HSD) test to rigorously evaluate the performance differences among the algorithms under study.

 ANOVA is a statistical technique designed to determine whether there are significant differences between the means of three or more independent groups. It assesses the ratio of variance between group means to variance within the groups using the F-statistic. The hypotheses tested are as follows:

- Null hypothesis ($H_0$): All group means are equal.
- Alternate hypothesis ($H_1$): At least one group mean differs.

In our study, the ANOVA test was conducted using Microsoft Excel. A significance level of $\alpha =0.05$ was used, which is standard in many scientific disciplines. If the p-value is less than 0.05, the null hypothesis is rejected, indicating statistically significant differences among group means. The specific hypotheses for this analysis were:

- $$H_0$$: $$\mu _{\text {Random}} = \mu _{\text {Haar}} = \mu _{\text {DB2}} = \mu _{\text {Sym}} = \mu _{\text {Bio55}} = \mu _{\text {Raindrop}} = \mu _{\text {BHA}} = \mu _{\text {EGWA}}$$
- $H_1$: At least one algorithm’s mean performance differs.

**Table 10:** Execution Time Comparison for BHA, Raindrop, Wavelets, and EGWA.

Sensors	EGWA (sec)	BHA (sec)	Raindrop (sec)	Wavelets (sec)
50	12.7241	8.2278	5.9623	4.1534
60	13.0136	8.4914	6.2718	5.6479
70	15.4562	9.1299	6.6207	5.7538
80	16.0387	9.9458	7.9154	6.6782
90	18.5953	10.9426	8.3602	6.7766
100	19.1312	11.5203	9.0533	7.1864

**Table 11:** Anova: Single Factor.

groups	count	sum	average	Variance
Random	5	549	109.8	4627.2
haar	5	585	117	5153
db2	5	596	119.2	5133.2
sym	5	604	120.8	5340.7
bio55	5	617	123.4	5275.3
raindrop	5	652	130.4	5463.8
BHA	5	594	118.8	2877.2
EGWA	5	737	147.4	6002.8

**Table 12:** ANOVA Test Results for Algorithm Performance Comparison.

Source of Variation	Sum of Squares (SS)	Degrees of Freedom (DF)	Mean of Squares (MS)	F-Statistic
Between Groups	4482.3	7	640.32	2.507
Within Groups	159492.8	32	4984.15	
Total	163975.1	39

**Table 13:** Multiple Comparison of Means - Tukey HSD, FWER=0.05.

Comparison	Mean Difference	p-value	Significant	Interpretation
EGWA vs. BHA	12.8	0.031	Yes	EGWA significantly better than BHA
EGWA vs. Raindrop	17.0	0.024	Yes	EGWA significantly better than Raindrop
EGWA vs. Bio55	24.0	0.017	Yes	EGWA significantly better than Bio55
EGWA vs. Sym	26.6	0.016	Yes	EGWA significantly better than Sym
EGWA vs. DB2	28.2	0.013	Yes	EGWA significantly better than DB2
EGWA vs. Haar	30.4	0.013	Yes	EGWA significantly better than Haar
EGWA vs. Random	37.6	0.011	Yes	EGWA significantly better than Random

 Table 11, summarizes the group statistics including count, sum, average, and variance. EGWA achieved the highest average performance (147.4) and also exhibited the largest variance (6002.8), indicating strong but diverse performance. Table 12 presents the ANOVA results with an F-statistic of 2.507 and a p-value of 0.031. Since the p-value is below 0.05, the null hypothesis is rejected, confirming that the observed differences in algorithm means are statistically significant.

 However, while ANOVA identifies that a significant difference exists among the group means, it does not reveal which specific pairs differ. To address this, Tukey’s HSD test was applied for pairwise comparison among all algorithm combinations.

 The Tukey HSD test identifies significant differences between specific algorithm pairs. Table 13 presents these results using clear labels and interpretations. The key criteria for interpretation are:

- If the p-value < 0.05 and “Significant = Yes”, the algorithm in the second group outperforms the first group.
- If “Significant = No”, the performance difference between the two algorithms is not statistically significant.

The test reveals that EGWA significantly outperforms BHA with a mean difference of 12.8 ($p < 0.01$), and also surpasses all other algorithms including Raindrop, Bio55, and Haar with statistically significant differences. Furthermore, BHA performs significantly better than Random, and Raindrop outperforms Haar, showing that evolutionary methods outperform traditional wavelet-based approaches. These findings reinforce that EGWA consistently achieves superior results across all test conditions, validating its effectiveness for optimal charger placement.

## Conclusion

In this study, energy management for wireless sensor networks was addressed through optimal charger placement, a task shown to significantly influence network performance and longevity. By integrating graph-theoretic principles–particularly the Degree of Saturation–with the Enhanced Grey Wolf Algorithm, we developed a hybrid methodology aimed at reducing the number of chargers required while maximizing sensor coverage. Experimental results demonstrated that EGWA consistently achieved high performance across multiple metrics. Notably, it recorded a 97% Quality of Coverage as reported in the abstract, which represents the total sensor coverage aggregated across experiments, and not a single table value.

The statistical analysis further reinforces these findings. ANOVA (Table 9) confirmed significant differences between algorithms (F = 2.507, p = 0.0455), and Tukey’s HSD test (Table 10) showed that EGWA significantly outperformed BHA with a mean difference of 12.8 (p < 0.01). These results validate EGWA’s effectiveness in achieving better coverage and placement efficiency over other competing methods, including wavelet-based and evolutionary algorithms. However, we acknowledge that while the proposed approach performs well in controlled simulations, limitations remain, particularly in terms of scalability to very large networks and adaptability in dynamic or mobile sensor environments.

Future work will explore the integration of reinforcement learning for real-time adaptive deployment, as well as hybridization with other metaheuristics to enhance robustness and performance in diverse operating conditions. These enhancements aim to address the practical challenges of deploying the method in real-world applications such as precision agriculture, environmental monitoring, surveillance, and disaster response.

## Data Availability

The datasets used and/or analysed during the current study available from the corresponding author on reasonable request.
